# A Case of Stricture of the Urethra, with Effusion of Urine into the Scrotum, Sloughing and Loss of a Testis; and a Case of Epilepsy in Which Trephining Was Resorted to

**Published:** 1844-05

**Authors:** William M. Yandell

**Affiliations:** Benton, Miss.


Art. III.—*A Case of Stricture of the Urethra, with effusion of urine into the Scrotum, sloughing and loss of a Testis; and a Case of Epilepsy in which trephining was resorted to.* By William M. Yandell, M.D., of Benton, Miss.

A negro man, aged about 50 years, had labored under a stricture of the urethra for fifteen years, during which time he had been attended by several physicians, who resorted to the use of the bougie without affording him relief. The stricture continued to increase, and at length the obstruction became so great, that the urine escaped by stillicidium. During last autumn he had an attack of bilious remittent fever, and while laboring under this disease the urine made its way into the scrotum. When I was called to see him the scrotum had become immensely distended, being in bulk as large as the head of a six year old boy. It was also gangrenous to a considerable extent, and on examination it was discovered that one of the testes was affected with gangrene also. His general health had suffered seriously, and his condition was altogether deplorable. The urine having been drawn off by a free incision, the parts were repeatedly washed with a solution of the chloride of soda, and soothing poultices were applied. The testis, which showed indisputable marks of gangrene, was excised after a day or two, and the patient put upon the iodide of iron. He improved rapidly, and in a short time was going about. For the stricture, which was still remaining, unalleviated, I commenced the strenuous use of the bougie, beginning with the smallest size metallic one, and have just had the satisfaction of dismissing the patient cured.

I discharged, to-day, a case of still greater interest to me. A young man, 21 years of age, from the fall of the limb of a tree, which fractured the right parietal bone, became subject to epileptic fits, and placed himself under my care about a month since. The depression in the bone extended in length to an inch and a half, and was about an inch broad. The accident occurred seven years ago. The convulsions ensued soon after, and have followed him ever since, his mind, in the mean time, having become impaired, as is usual in that disease. The case appeared to me to be one of those in which a surgical operation afforded the only chance of recovery. I applied the trephine, in presence of my brother, Dr. Burton Yandell, four weeks ago, by which I removed, at first, a portion of the depressed bone. The inner surface of the bone was smooth and healthy, as was also the dura mater —pulsation of the brain scarcely perceptible. A probe being passed under the depressed bone which remained, two ounces of bloody serum escaped, but I was not able to discover any roughness of the bone, or any spiculæ growing from its surface upon the dura mater. I determined, however, to re-apply the trephine, and remove the remainder of the depressed cranium, so as to see the source of the fluid, and the condition of the dura mater at that point, as well as to relieve the brain from pressure. I proceeded cautiously, raising the bone with the elevator before the incision had been made complete all around. On lifting out the bone, a gush of blood, in a stream larger than a goose-quill, followed, and seemed to threaten the life of the patient, but was promptly restrained by pressure. It was soon discovered that complete paralysis of the left leg and arm had supervened, in consequence, perhaps, of the pressure used to control the hemorrhage. This symptom, however, is giving way under the use of friction and rubefacients, and I have no doubt will entirely disappear when the parts return to their natural state. His general health has become quite good; he has had no return of the convulsions up to this time, and his mind appears to be gaining in vigor. It is yet too early to predict what will be the final issue of the case, but I may remark that no untoward symptom now exists, and the promise is, that he will remain exempt from his distressing malady.

Benton, March 20, 1844.

